# Evaluation of double faced transverse preputial (onlay) island flap for hypospadias repair in pediatrics: a randomized controlled study

**DOI:** 10.1007/s11255-022-03217-1

**Published:** 2022-04-24

**Authors:** Mohammad Daboos, Khalid Hefney, Muhammad Abdelhafez Mahmoud, Ahmed Salama, Yousef Mohammed, Mohammed Hussein, Mohamed Abdelmaboud, Tharwat Hussein, Yasser Ashour, Samir Gouda

**Affiliations:** Pediatric Surgery Department, Al-Azhar University, Al-Houssain University Hospital, Darrasa, Cairo Egypt

**Keywords:** Double face, Onlay island flap, Inner preputial flap, Hypospadias in pediatrics

## Abstract

**Background:**

The preputial onlay island flap technique has been popularized for hypospadias repair as a result of offering a consistent combination of acceptable functional and cosmetic results. Like other techniques, urethrocutaneous fistulae and stricture continues to be the most common complications, in addition to other complications, which could be attributed to the compromise in flaps vascularity. Some authors describe a technique that resolves some of these problems by combining the unique benefits of the double faced preputial flaps. The aim of this study:- to evaluate double faced preputial onlay island flap technique for complications rate, outcomes of surgical procedure, and cosmetic results in comparison to transverse inner preputial flap technique.

**Patients and methods:**

This was a prospective randomized controlled study that included 68 patients with anterior, mid-penile, and posterior penile hypospadias, with shallow and narrow urethral plate of size less than 6 mm, who underwent single-stage repair using preputial flaps, conducted at the department of pediatric surgery (Al-Azhar University, Cairo, Egypt), between May 2019 and October 2021, to evaluate double faced transverse preputial onlay island flap technique. Thirty-four patients underwent double faced transverse preputial onlay island flap (group A) and another 34 patients underwent inner transverse preputial onlay island flap (control group) (group B). The follow-up period ranged from 12 to 26 months.

**Results:**

The overall complication rate was 20.5% (14 of 68 children). Complications developed in 5 cases (14.7%) in group A who underwent double face onlay island flap (2 glannular dehiscence, 1 penile rotation, 1 fistula, and 1 diverticulum), as opposed to 9 patients in group B (26.4%) who underwent transverse inner preputial flap (3 developed glannular dehiscence, 2 skin flap necrosis, 3 fistulae, and 1 diverticulum). After management of the complications, all patients had good surgical outcomes with satisfactory cosmetic results.

**Conclusion:**

Double faced transverse preputial onlay island flap is an alternative option to reconstruct narrow urethral plate hypospadias. So that double faced transverse preputial onlay island flap technique appears to achieve satisfactory surgical outcomes with lower complication rate.

## Introduction

The preputial onlay island flap technique has been popularized for hypospadias repair as a result of offering a consistent combination of acceptable functional and cosmetic results. Like other techniques, urethrocutaneous fistulae, urethral stricture, and recurrence, continue to be the common postoperative complications. All those complications can be attributed to insufficiency of flaps vascularity [1–6].

Some authors describe a technique that resolves some of these problems by combining the unique benefits of the double faced preputial flap to achieve successful repair with fewer complications and provide better cosmetic outcomes. [7–9]. In this study, we aimed to evaluate double faced preputial onlay island flap in hypospadias repair for incidence of complications, outcomes of surgical procedure, and cosmetic results.

## Patients and methods

This was a prospective randomized controlled study conducted at Al-Houssain and Sayed Galal University Medical Centers (department of pediatric surgery, Al-Azhar University) in the period from May 2019 to October 2021, to evaluate double faced transverse preputial onlay island flap. Sixty-eight patients fulfilled the required criteria were included in the study. The subjects were limited to the patients diagnosed as anterior, mid-penile, or posterior penile hypospadias with shallow and narrow urethral plate measuring less than 6 mm, without or with mild penile curvature of less than 30º after degloving of the penis. Patients were randomly allocated to underwent one of two surgical techniques (Group A and B) using closed sealed envelope method. Thirty-four patients underwent hypospadias repair using double faced transverse preputial onlay island flap (Group A). And the remaining 34 patients underwent inner transverse preputial onlay island flap repair (Group B).

Patients with wide urethral plate suitable for Tubularized Incised Plate (TIP) urethroplasty, other types of hypospadias as penoscrotal, scrotal, or perineal hypospadias, patients with moderate or severe penile curvature and recurrent cases were excluded from this study.

All operations were done by the same surgical team. Our institutional review board approval was obtained (IRB: 00,012,368–19-05–009) and the study was registered at ClinicalTrials.gov (ID: NCT05144659). All procedures were performed after signed written informed consent by the parents. Main outcome measurements included: postoperative complications, surgical outcomes, and cosmetic results, all were assessed by Hypospadias Objective Scoring Evaluation (HOSE) questionnaire at follow-up visits.

### Surgical procedures

**Fig. 1:**
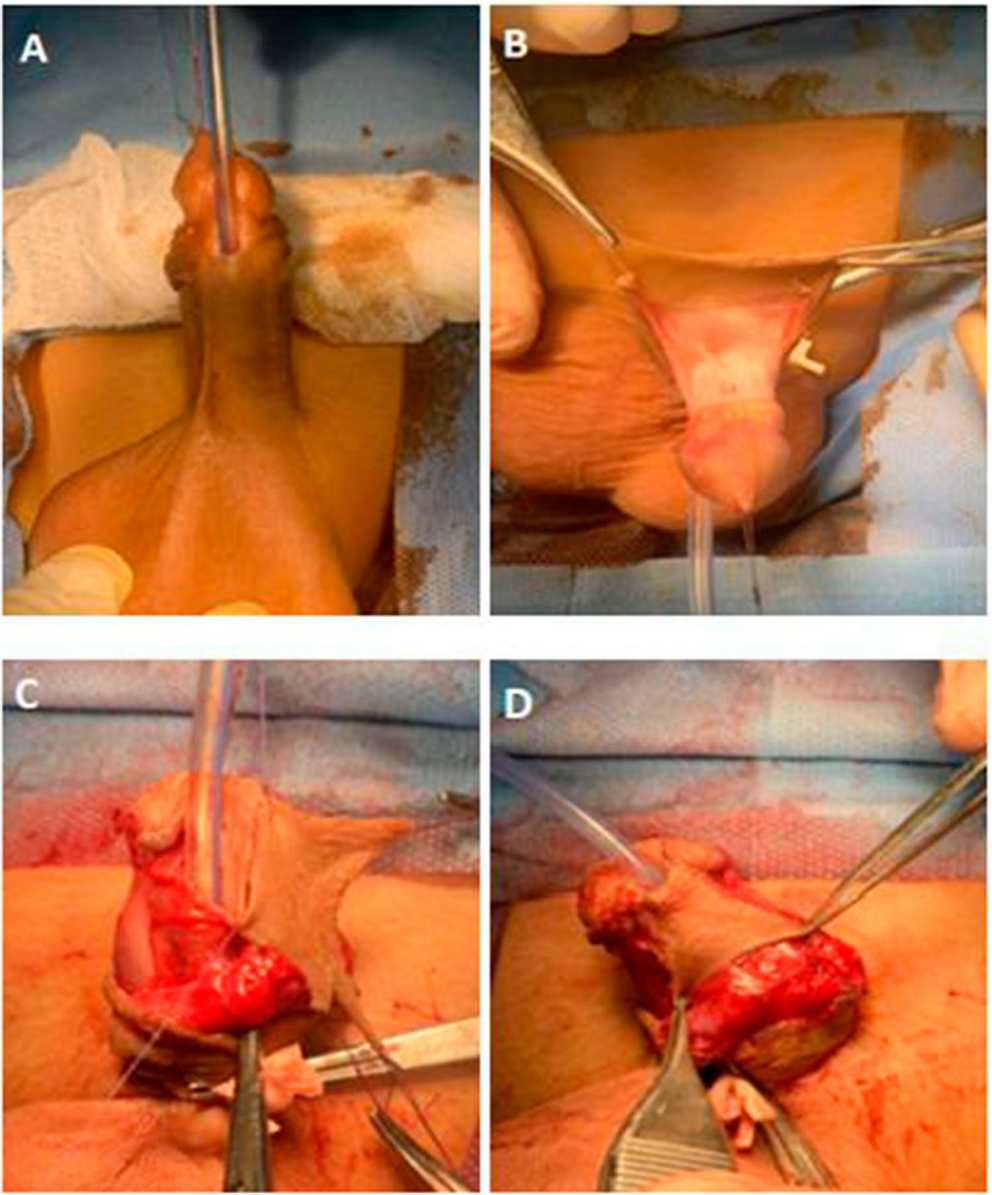
Double faced transverse preputial onlay flap. **A** Anterior penile hypospadias **B** Dissection of double faced transverse preputial flap **C** This inner (onlay) flap was sutured to the urethral plate with running 6-zero vicryl sutures **D** The outer preputial flap was trimmed to cover the ventral shaft skin defect

**Fig. 2:**
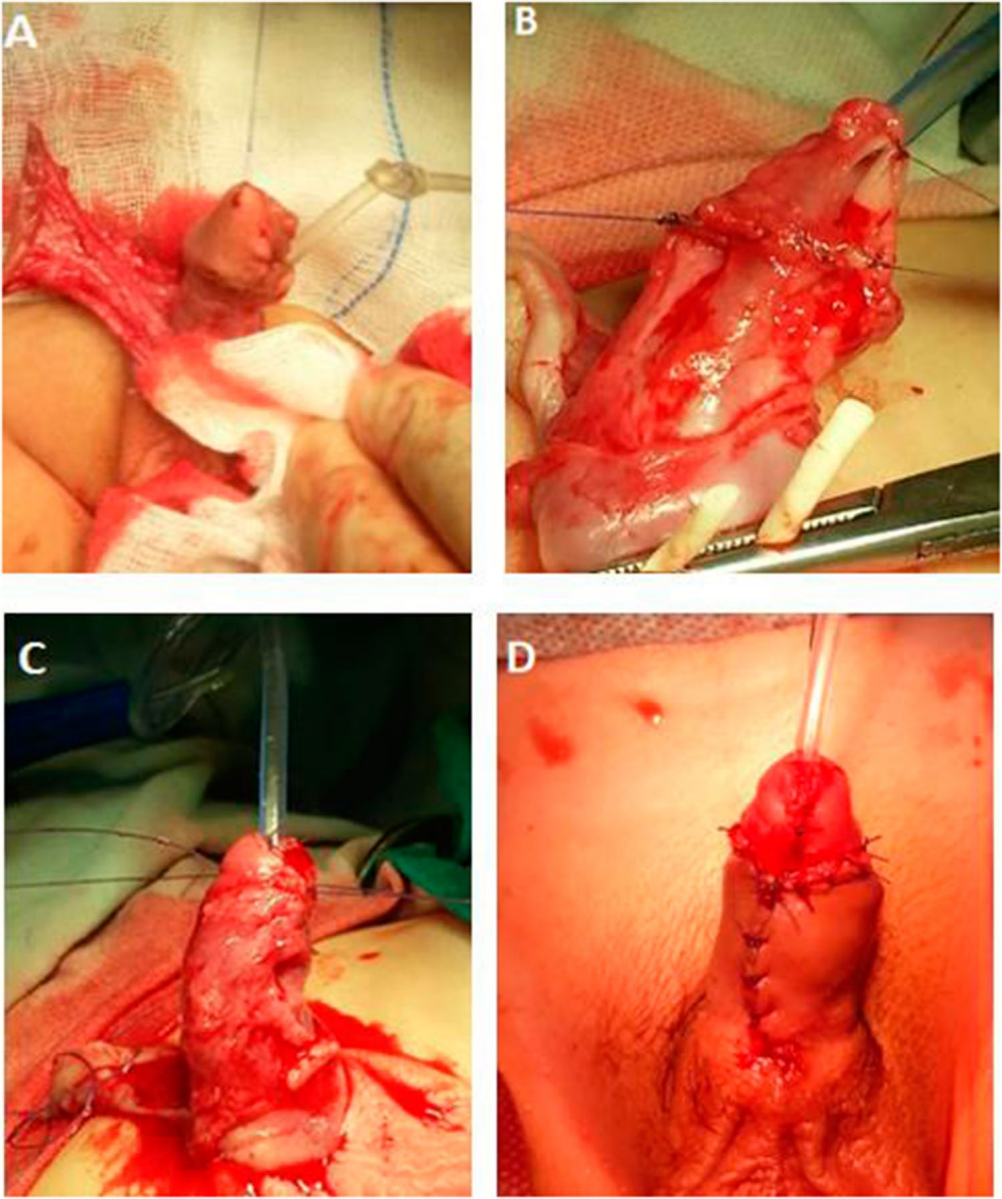
Inner transverse preputial flap. **A** Dissection of transverse preputial flap. **B** the flap transposed ventrally and sutured. **C** Glannular wings closure and anchoring dartos fascia over the repair **D** The skin was sutured to remaining skin on both lateral sides (ventral skin closure) using Bayers flap

Under general anesthesia, after draping, a traction suture was placed in the glans and the urethral plate is defined by 2 parallel incisions, which were curved proximally to the original meatus. An incision was made circumferentially 2–3 mm. proximal to the coronal sulcus. The penile skin was degloved along Buck's fascia proximally down to penopubic junction. All fibers bands around the corpus spongiosum were excised to correct any curvature which was present. Artificial erection test was performed to identify the site and degree of ventral penile curvature which was measured using digital goniometer. In cases with residual mild curvature, dorsal corporeal plication was done using 2 midline 5/0 silk sutures at 12 o’clock, (nerve-free zone). After curvature correction, the urethral plate was outlined into the glans and lateral glans flaps were prepared. **In group (A)**: a total preputial flap was created by a transverse incision at the junction of the penile shaft skin and the outer layer of the prepuce, then dissection of its vascular pedicle to the penopubic angle was done (Fig. 1A, B). Then, the flap was transposed ventrally by button-hole method and rotated 90 degrees. The inner (onlay) flap was measured to be 10 mm, then outlined and sutured to the urethral plate by running 6-zero vicryl sutures (Fig. 1C), over 8Fr Nelaton catheter with inversion of the flap edges. The onlay flap was trimmed as per the situation demanded. The urethral meatus was constructed by suturing its free distal border to the glans with interrupted 6-zero vicryl sutures, and the neomeatus was fashioned as oval shape to avoid meatal stenosis. Then, the glannular flaps were closed ventrally with 6-zero vicryl sutures, the dorsal shaft skin was sutured to the coronal skin, and the outer preputial flap was trimmed to cover the ventral shaft skin defect (Fig. 1D). **In group (B)**: a flap of appropriate width and length, pedicled on Dartos fascia, was harvested from the inner preputial skin with dissection of outer layer of preputial flap and dorsal penile skin up to penopubic angle. Then, the flap was rotated ventrally after buttonholing its base. The flap was assessed and edges were trimmed. Then, it was sutured with the urethral plate over 8 Fr. Nelaton catheter using 6/0 Vicryl suture with inversion of edges (Fig. 2A, B), and the neomeatus fashioned as oval shape to avoid meatal stenosis. The glannular flaps were closed ventrally with 6-zero vicryl sutures, and dartos fascia was fixed over the repair (Fig. 2C), then dorsal shaft skin was sutured to the coronal skin and ventral skin closure was done (Fig. 2D).

### In both groups

Postoperative prophylactic antibiotics (Amoxicillin 100 mg/kg/day), analgesics (paracetamol 15 mg/kg/dose), and antispasmodics (oxybutynin) were given till discharge. Urethral catheter was left in diaper for 8–10 days. Follow-up was made at 1, 3, 12, and 24 months, for incidence of any complications. HOSE score questionnaire was used for the evaluation of outcomes at post-operative visits.

### Statistical analysis

Statistical analysis was performed using statistical package for social sciences (SPSS) version 23.0, IBM Corp, IBM SPSS Statistics for Windows, Armonk, NY, USA. For qualitative data, chi-square test (X^2^) was used to compare between the two groups and independent-samples t-test of significance was used when comparing between two means. The significance level was set at *P* < 0.05.

## Results

The present study included 68 children. Their age at repair ranged from 12 months to 7 years (mean: 3.2 years) in group A, and ranged from 10 months to 7.5 years (mean: 3.3 years) in Group B. Follow-up period ranged between 12 and 26 months. Thirty-four patients underwent single-stage hypospadias repair, using double faced preputial onlay island flap (group A), included (15 anterior penile, 16 mid-penile, and 3 cases with posterior penile hypospadias), and 34 patients underwent inner preputial onlay island flap (group B), included (16 anterior penile, 16 mid-penile, and 2 cases with posterior penile hypospadias).

The overall complication rate was 20.5% (14 of 68 children). Complications developed in 5 cases (14.7%) in group A who underwent double faced onlay island flap (2 cases of glannular dehiscence, 1 case of penile rotation, 1 case of urethrocutaneous fistula, and 1 case of diverticulum), while 9 patients developed complications in group B (26.4%) who underwent transverse inner preputial onlay island flap (3 cases developed glannular dehiscence, 2 cases of skin flap necrosis, 3 cases of urethrocutaneous fistulae and 1 case of diverticulum). The difference between the complications rate in both groups was statistically significant (*p*-value > 0.05) Table 1.

**Table 1:** Complication rate of double face preputial onlay island flap (Group A) and inner preputial onlay island flap (Group B)

Complications	Group A (*n* = 34)	Group B (*n* = 34)	*x*^*2*^	*p-value*
Urethrocutaneous fistula	1 (2.9%)	3 (8.8%)	3.935	0.047*
Flap necrosis	0 (0.0%)	2 (5.8%)	4.201	0.020*
Glanular dehiscence	2 (5.8%)	3 (8.8%)	2.207	0.137
Penile rotation	1 (2.9%)	0 (0.0%)	0.986	0.321
Urethral diverticulum	1 (2.9%)	1 (2.9%)	0.000	1.000
Total complications	5 (14.7%)	9 (26.4%)	6.924	< 0.031*

The difference between the complication rates in both groups was statistically significant.

Using: chi-square test; *p*-value > 0.05 NS; *p-value < 0.05 S; **p-value < 0.001 HS

Postoperatively, all patients were submitted to Hypospadias Objective Scoring Evaluation (HOSE) to evaluate the outcomes regarding the incidence of complications and cosmetic results Table 2.

**Table 2:** HOSE questionnaire variables for both groups

Variable of HOSE	HOSE score	No of patients in Group A (*n* = 34)	No of patients in Group B (*n* = 34)
*Meatal location*			
Distal glannular	4	32	29
Proximal glannular	3	2	2
Coronal	2	0	1
Penile shaft	1	0	2
*Meatal shape*			
Vertical slit	2	11	13
Circular	1	23	21
*Urinary stream*			
Single stream	2	31	29
Sprayed	1	3	5
*Erection*			
Straight	4	34	34
Mild angulation	3	–	–
Moderate angulation	2	–	–
Sever angulation	1	–	–
*Fistula*			
None	4	33	31
Single distal	3	1	1
Single proximal	2		2
Multiple or complex	1	–	–

In group A, the score ranged between 12 and 16, while in group B, the score ranged between 10 and 16.

HOSE score, defined by Holland et al. in 2001

The postoperative HOSE score in Group A ranged between 12 and 16 and the mean was (14.9 ± 1.1), while the mean postoperative HOSE score in Group B was (12.4 ± 1.7), ranging between 10 and 16. The difference between two groups regarding HOSE score was statistically significant Table 3.

**Table 3:** The difference between HOSE score for both groups was statistically significant

Mean HOSE score	Group A (*n* = 34)	Group B (*n* = 34)	*P*-value
Mean ± SD	14.9 ± 1.1	12.4 ± 1.7	< 0.001**

Using: independent sample *t*-test; *p*-value < 0.001 HS

**Fig. 3:**
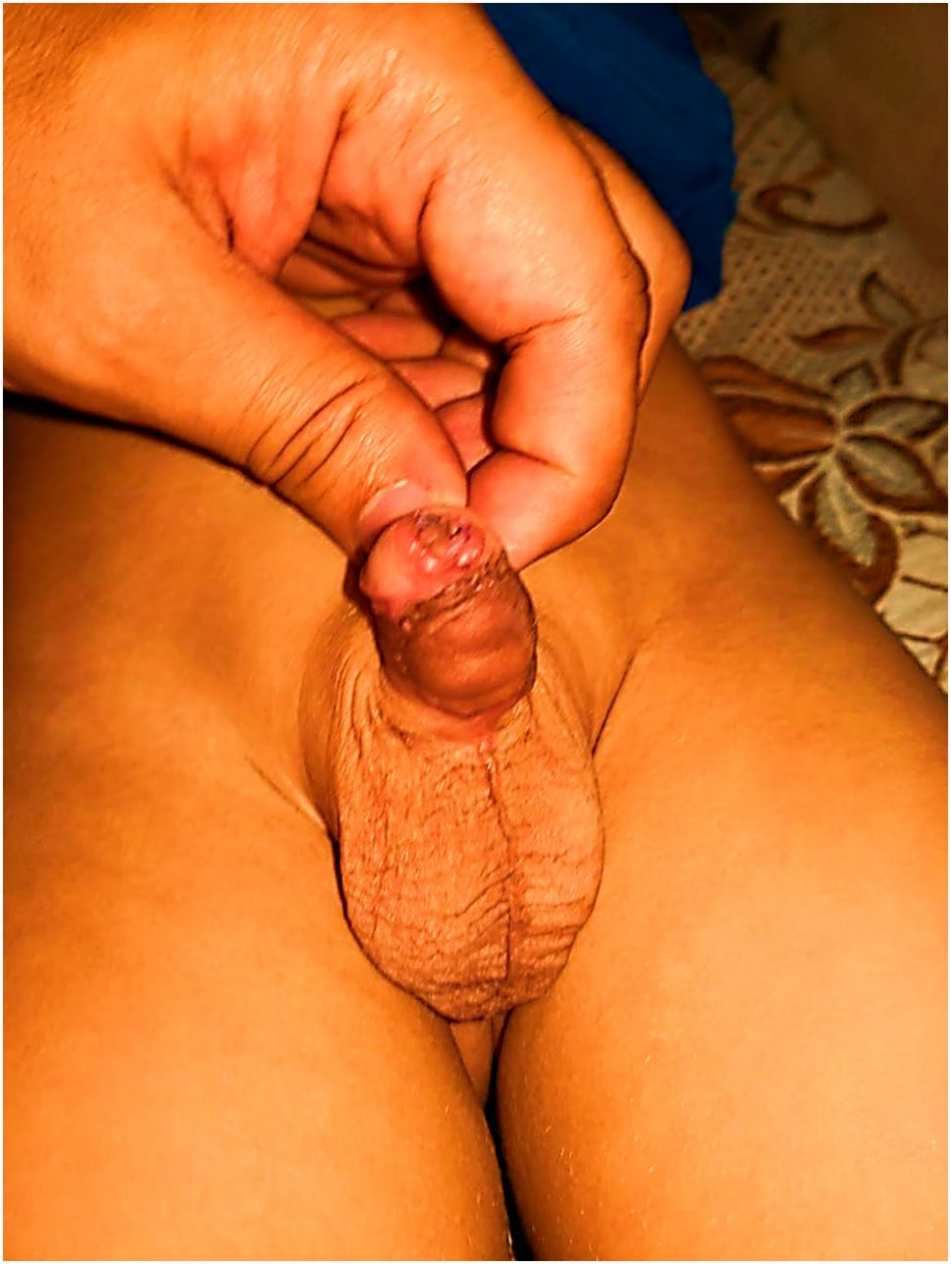
Late pictures of double faced onlay island preputial flap technique

Four children underwent fistula repair at a second operation. The parents of the child with penile rotation refused the second operation. The children with glans dehiscence were treated successfully in a second repair. Two children with flap necrosis were treated successfully by tubularized urethroplasty and another 2 cases of urethral diverticulum were managed successfully by reduction urethroplasty. After management of the complications, all patients had good surgical outcomes and satisfactory cosmetic results (Fig. 3).

## Discussion

The concept of a vascularized preputial island flap was introduced by *Hook in 1896* [10]. *Asopa and colleagues* developed the first very effective use of inner preputial skin for a substitution urethroplasty [8]. Duckett developed this by describing a transverse island tube repair in 1980. By 1980s gradually, it became recognized that most penile curvatures in hypospadias is due to the skin and subcutaneous tissue asymmetry. So that after correction of the curvature, the urethral plate could be safely incorporated into a hypospadias repair [11–13].

Many authors suggested that the dissection of the vascularized pedicle flaps from dorsal preputial tissue may affect the vascularity and increase the complication rate and also showed that transferring the flap with its skin covering appears to achieve better results [7–9, 14].

Penoscrotal and the most proximal hypospadias are usually associated with moderate or severe penile curvature in about 68–70%, so only 11–24% of surgeons preferred to do onlay island flap in such cases, On the other hand, more than 50% of hypospadias surgeons preferred to do staged repair [15, 16]. Braga et al*.* in their study submitted 40 patients with proximal hypospadias for onlay island flap urethroplasty. As a result, complications occurred in 45% of patients, and they reported that recurrent ventral penile curvature was more frequent. So, in this study, we preferred to exclude the cases with penoscrotal or the most proximal hypospadias and cases with moderate or severe penile curvature, to provide better surgical outcomes and avoid recurrence of penile curvature. [17].

In this study, we agreed with *Gonzalez *et al. *1996* in their series, emphasizing that double faced onlay preputial flap technique has resulted in satisfactory functional, cosmetic outcomes, and low complication rates [7].

*Abdelbaset *et al. *2017 and Daboos *et al. *2020* suggested that the dissection of the vascularized pedicle flap from dorsal preputial tissue may affect the vascularity and increase the complication rate, and also showed that transferring the tube or flap with its skin covering appears to achieve better results [9, 14].

*In the present study*, we performed a one-stage procedure using two well-established techniques to repair anterior and mid-penile hypospadias in 68 cases without or with mild penile chordee, categorized into two groups. The first group (group A) underwent double face preputial onlay island flap procedure. While second group (group B) underwent inner transverse preputial onlay island flap procedure. Five cases (14.7%) developed Complications in (group A), while 9 patients (26.4%) developed complications in (group B), with statistically significant difference of complications between two groups. *Barroso *et al. *2000* reported that complications requiring reoperation occurred in 12 patients (25%) in 47 children who underwent double faced onlay island flap repair. In their series, they have higher incidence of urethrocutaneous fistulae (8 cases i.e. about 17%), while in our series, we have one case of urethocutaneous fistula (2.9%). The lower rate of fistulae in our study may be due to meticulous dissection of preputial flap with preservation of its vascularity, adequate and integrated closure, in addition to the dorsal non dissected skin flap covering, provided more protection and securing for the suture lines. Also, they have higher rate of urethral diverticulum (about 4 cases). So, proper measuring of the flaps width and length leads to lower incidence of diverticulum in our series [18].

*In the current study,* we have results nearly similar to those of *Chin *et al. *2001,* regarding the urethrocutaneous fistula and glannular disruption, after hypospadias repair using a double faced onlay island flap performed in 15 patients with middle and posterior penile hypospadias. Postoperative complications with *Chin* et al. occurred in 2 patients: 1 developed a subcoronal fistula and 1 had dorsal skin necrosis and suture disruption of the glannular wings. The overall complication rate was 13% and they reported that method provides a well-vascularized ventral skin cover and reduces the area of avascular dorsal skin [19].

*El dahshoury *et al. *2013* adopted the technique of double faced onlay island flap in 160 cases of distal and mid-shaft hypospadias and they had similar incidence of urethral diverticulum, penile rotation, and one case of glannular dehiscence in spite of they had harvested the outer preputial layer as a triangular flap sutured to the ventral aspect of the proximal non-approximated glannular wings, to avoid closure of the glannular wings under tension. In this study, we also agree with *El dahshoury *et al., in that penile torsion was recorded in only one case due to insufficient dissection of penile skin down to the penopubic angle [20].

Outcomes of hypospadias repair can be analyzed using both objective and subjective criteria. Objective criteria include functional evaluation of micturition by uroflowmetry, which is difficult to interpret in children as its profile is often abnormal even if reconstruction is satisfactory, may be due to child cooperation difficulties. [9] Objective evaluation of urinary function using uroflowmetry could not be done in this study due to difficulty in cooperation, as most of cases were before toilet training age.

By reviewing the literature, few studies had adopted HOSE score for assessment of postoperative outcome after onlay island flap procedure. In spite of HOSE score has been validated as a pediatric objective scoring system for evaluating the outcomes of hypospadias repair, as it incorporates the outcomes of meatal location, shape, urinary stream, the straightness of erection, and any urethral fistula [21].

In their original series for application of HOSE score, *Holland *et al. *2001*, had used HOSE score for assessment of postoperative outcomes of different hypospadias repair techniques (including onlay island flap for 11 patients from a total of 20 patients) for repair of anterior and middle hypospadias. The HOSE assessment gave a total score of 12–16 [21]. *Seibold *et al. *2010* showed that the mean HOSE score was 15 (range 12–16) out of a maximum score of 16, with a score of 14 or greater defined as excellent. Ninety-three patients (94%) reached the maximum of 16 points. Six patients (6%) reached 12–15 points. Hence, 96 patients (97%) had an excellent surgical outcome [22].

A score of 14 or more (maximum score of 16) was suggested by *Liu MM *et al. *2015* in their series on different techniques to infer an acceptable outcome [23]. We came from the same way as the previous studies; because we found 29 cases (about 85%) in group A and 25 cases (about 74%) in group B achieved more than 14 points. And the least score ranged between 10 and 12 points were reported in both groups. At the end of this study, we can say that our series is the only one that applied HOSE score for assessment of Double Faced Onlay Island Flap.

Limitations *of this study included: relatively small number of cases, due to COVID-19 pandemic restriction, and relatively short follow-up period, so a larger number of cases with a longer follow-up period is recommended by the authors.*

## Conclusion

Double faced transverse preputial onlay island flap is an alternative option to reconstruct narrow urethral plate hypospadias. So that double faced transverse preputial onlay island flap technique appears to achieve satisfactory surgical outcomes with lower complication rate.
